# Anti-Fibrotic Actions of Interleukin-10 against Hypertrophic Scarring by Activation of PI3K/AKT and STAT3 Signaling Pathways in Scar-Forming Fibroblasts

**DOI:** 10.1371/journal.pone.0098228

**Published:** 2014-05-30

**Authors:** Jihong Shi, Jun Li, Hao Guan, Weixia Cai, Xiaozhi Bai, Xiaobing Fang, Xiaolong Hu, Yaojun Wang, Hongtao Wang, Zhao Zheng, Linlin Su, Dahai Hu, Xiongxiang Zhu

**Affiliations:** Department of Burns and Cutaneous Surgery, Xijing Hospital, Fourth Military Medical University, Xi’an, China; University Hospital Hamburg-Eppendorf, Germany

## Abstract

**Background:**

The hypertrophic scar (HS) is a serious fibrotic skin condition and a major clinical problem. Interleukin-10 (IL-10) has been identified as a prospective scar-improving compound based on preclinical trials. Our previous work showed that IL-10 has anti-fibrotic effects in transforming growth factor (TGF)-β1-stimulated fibroblasts, as well as potential therapeutic benefits for the prevention and reduction of scar formation. However, relatively little is known about the mechanisms underlying IL-10-mediated anti-fibrotic and scar-improvement actions.

**Objective:**

To explore the expression of the IL-10 receptor in human HS tissue and primary HS fibroblasts (HSFs), and the molecular mechanisms contributing to the anti-fibrotic and scar-improvement capabilities of IL-10.

**Methods:**

Expression of the IL-10 receptor was assessed in HS tissue and HSFs by immunohistochemistry, immunofluorescence microscopy, and polymerase chain reaction analysis. Primary HSFs were treated with IL-10, a specific phosphatidylinositol 3 kinase (PI3K) inhibitor (LY294002) or a function-blocking antibody against the IL-10 receptor (IL-10RB). Next, Western blot analysis was used to evaluate changes in the phosphorylation status of AKT and signal transducers and activators of transcription (STAT) 3, as well as the expression levels of fibrosis-related proteins.

**Results:**

HS tissue and primary HSFs were characterized by expression of the IL-10 receptor and by high expression of fibrotic markers relative to normal controls. Primary HSFs expressed the IL-10 receptor, while IL-10 induced AKT and STAT3 phosphorylation in these cells. In addition, LY294002 blocked AKT and STAT phosphorylation, and also up-regulated expression levels of type I and type III collagen (Col 1 and Col 3) and alpha-smooth muscle actin (α-SMA) in IL-10-treated cells. Similarly, IL-10RB reduced STAT3/AKT phosphorylation and blocked the IL-10-mediated mitigation of fibrosis in HSFs.

**Conclusion:**

IL-10 apparently inhibits fibrosis by activating AKT and STAT3 phosphorylation downstream of the IL-10 receptor, and by facilitating crosstalk between the PI3K/AKT and STAT3 signal transduction pathways.

## Introduction

Scarring is an expected result of wound healing [1], [2]. However, in some individuals the wound healing process leads to development of a fibrotic hypertrophic scar (HS) characterized by raised, red and inflexible skin tissue. Such scars can cause serious functional and cosmetic problems and also result in psychological and physical suffering [3]–[6]. The incidence of HS ranges from 40–70% following surgery, and up to 91% following burn injury [7]. However, there is currently no effective therapy for HS, in part because the underlying mechanisms of HS progression are poorly understood [7], [8].

Interleukin 10 (IL-10) was first described as a cytokine-synthesis inhibitory factor with anti-inflammatory functions [9], [10]. IL-10 is expressed by a variety of mammalian cell types, including macrophages, monocytes, Th2 cells, B cells, mast cells, dendritic cells, regulatory T cells and keratinocytes [11], [12]. Although IL-10 is classified as a Th2-type cytokine, the molecule suppresses a broad range of inflammatory responses and is important for the maintenance of homeostasis during infection and inflammation [10]. As a major immunosuppressive and anti-inflammatory factor, IL-10 also plays a pivotal role in wound healing [11], [13]. In pathological scars, IL-10 exerts regulatory actions against the recruitment and differentiation of inflammatory cells and the production of pro-inflammatory cytokines [14]–[17].

IL-10 is now regarded as a promising new therapeutic agent for scarring [17]–[19]. Based on preclinical studies, IL-10 is presumed to reduce skin scarring by the following mechanisms: (1) modulation of inflammatory cell recruitment and differentiation, together with down-regulation of the production and secretion of pro-inflammatory cytokines [14], [15], [17]; (2) attenuation of extracellular matrix (ECM) production [16], [20] and augmentation of ECM breakdown via up-regulation of proteolytic enzymes [19], [21], [22]; and (3) down-regulation of transforming growth factor (TGF)-β1 expression and the ensuing fibrosis [19], [23], [24]. Indeed, our recent study [6] identified a protective role for IL-10 against TGF-β1-induced fibrosis in dermal fibroblasts and highlighted the potential therapeutic impact of IL-10 for scar improvement. The results showed that IL-10 down-regulated collagen expression, up-regulated matrix metalloproteinase (MMP) 1 and MMP8 expression, and repressed the transformation of fibroblasts into alpha-smooth muscle actin (α-SMA)-positive myofibroblasts, leading to the degradation of abnormally deposited ECM components and a decrease in excessive ECM secretion. However, much remains unknown about the molecular mechanisms of IL-10 against skin fibrosis and scar formation.

IL-10 is generally thought to exert its biological actions by interacting with a specific cell membrane receptor and by activating the signal transducers and activators of transcription (STAT) 3-mediated signaling pathway [24]–[26]. Specifically, IL-10 dimerizes and binds to a cell-bound cytokine receptor, the IL-10 receptor, which is composed of two molecules of IL-10 receptor α-chain (IL-10Rα) and two molecules of accessory IL-10 receptor β-chain (IL-10Rβ). In contrast to IL-10Rα, which is unique to the IL-10 receptor, IL-10Rβ is shared by the receptors for several other cytokines, such as IL-22, IL-26, λ-interferon (λ-IFN), IL-28A/B and IL-29 [25], [26].

HS is a major skin fibrotic disorder characterized by excessive cell proliferation and deposition of ECM components [27]–[29], particularly type I and type III collagen (Col 1 and Col 3). HS is also associated with the transformation of fibroblasts into myofibroblasts [1], [6], [30], [31]. HS formation can provoke substantial obstacles to tissue growth, function, movement, and aesthetics, and as noted above, creates serious psychological and physical issues for HS patients [3]–[5], [32]–[35].

Fibroblasts undoubtedly represent one of the most important types of effector cell responsible for HS formation [1], [36], [37]. To further explore the molecular mechanisms underlying the anti-fibrotic properties of IL-10 during HS development and progression, we first investigated the expression of IL-10Rα and fibrosis-related proteins in HS tissue and primary cultured HS fibroblasts (HSFs). Next, we investigated changes in the phosphorylation status of AKT and STAT3 in IL-10-treated HSFs with or without a specific phosphatidylinositol 3 kinase (PI3K) inhibitor, LY294002, or a function-blocking antibody against the IL-10 receptor, IL-10RB. The current results clearly indicate that IL-10 inhibits fibrosis in HSFs by activating crosstalk between PI3K/AKT and STAT3 signal transduction pathways.

## Materials and Methods

### Collection and Processing of HS Tissue

HS and corresponding normal dermal skin (NS) tissues were obtained from patients who had undergone surgical excision at Xijing Hospital (Xi’an, China). Before surgery, the patients were informed about the purpose and procedure of the study and voluntarily agreed to provide skin tissue. Written consent was obtained from all participants, and all protocols were approved by the Ethics Committee of Xijing Hospital affiliated to the Fourth Military Medical University. After collection, skin samples were cut into four portions. One portion was preserved in 10% buffered formalin solution for histopathological analysis, the second in liquid nitrogen for the preparation of total RNA, and the third in liquid nitrogen for the preparation of total protein lysate. The fourth portion was used for the isolation and culture of fibroblasts, as described below.

### Evaluation of HS in Biopsy Tissue

The excessive deposition of collagen is a hallmark of HS [1], [30], [31]. The selection of the HS samples used in this study was primarily based on strict clinical criteria (i.e., locally thickened, raised, red, hard, inelastic and pruritic tissue), followed by confirmation of the histologic characteristics of the collagen fibers by routine hematoxylin and eosin (H&E) staining and Masson’s trichrome staining. In addition, the molecular verification of abnormal collagen expression/deposition in the HS tissue was performed by real-time quantitative polymerase chain reaction (RT-qPCR) and Western blot analysis. Detailed methods and data collection are described below.

### Histopathology, Immunohistochemistry, and Immunofluorescence Analysis

Biopsy tissues were fixed in 10% buffered formalin, embedded in paraffin blocks, and cut into 3-µm-thick tissue sections. One of the tissue sections was used for routine H&E staining and another was used as previously described for Masson’s trichrome staining analysis of collagen fibers [6], [38], [39]. The histologic characteristics of the collagen fibers in the samples were observed under a microscope.

For immunohistochemistry, sections were dewaxed, and endogenous peroxidase activity was quenched with 3% hydrogen peroxide for 15 min. Sections were then blocked with normal goat serum for 30 min to eliminate non-specific binding and incubated with a primary monoclonal antibody against IL-10Rα (1∶100 dilution; Santa Cruz Biotechnology, Santa Cruz, CA, USA) at 4°C overnight. The next day, sections were subjected to immunostaining with a SP-9000 Histostain™ kit (ZSGB, Ningbo, China). Briefly, sections were incubated with biotinylated secondary antibody, followed by signal amplification with streptavidin-biotin-horseradish peroxidase and staining with diaminobenzidine (DAB). Finally, the sections were counterstained with hematoxylin. An isotype-matched IgG was used as the negative control for each immunostaining procedure.

For immunofluorescence, sections were dewaxed, blocked, incubated with primary antibodies and then treated with the corresponding fluorescein isothiocyanate (FITC) or Cy3-conjugated secondary antibody. For cultured fibroblasts, immunofluorescence was performed as previously reported [6], [39]. In brief, cells were grown on coverslips for 48 h until they reached 70–80% confluence, followed by fixation in 4% formaldehyde for 30 min. After washing with phosphate buffered saline (PBS), the cells were permeabilized with 0.1% Triton X-100 for 10 min at room temperature and then blocked with 1% bovine serum albumin to eliminate non-specific binding. Samples were incubated at room temperature for 1 h with mouse monoclonal antibody against IL-10Rα (1∶500 dilution; Santa Cruz Biotechnology) or with rabbit monoclonal antibody against α-SMA (1∶100 dilution; Epitomics, Burlingame, CA USA). Samples were then treated with Cy3-conjugated goat anti-rabbit or goat anti-mouse secondary antibody (1∶100 dilution; CWBIO, Beijing, China) at 37°C for 1 h. Finally, the samples were stained with 4′,6′-diamidino-2-phenylindole (DAPI).

### Cell Culture and Treatment

Cell culture was performed as previously reported [6], [28], [39]. Briefly, HS and NS tissues were minced and incubated in a solution of collagenase type I (0.1 mg/ml; Sigma, St. Louis, MO, USA) at 37°C for 2.5 h to isolate fibroblasts. Fibroblasts were then pelleted and grown in Dulbecco’s Modified Eagle Medium (Gibco, Grand Island, NY, USA) supplemented with 10% fetal calf serum (Gibco), 100 U/ml penicillin, and 100 U/ml streptomycin. Cells were incubated at 37°C in a 5% (v/v) CO_2_-humidified atmosphere. All experiments were performed with passage 3–5 cells.

Confluent (70–80%) fibroblasts were incubated for 12–16 h in serum-free medium and then treated with IL-10 (10 ng/ml; PeproTech Inc., Rocky Hill, NJ, USA), an IL-10 receptor function-blocking antibody (IL-10RB, 1∶500 dilution, Santa Cruz Biotechnology), or the PI3K inhibitor LY294002 (50 µM; Beyotime Biotech, Nanjing, China) for 30 min, 24 h, or 48 h. The cells were then subjected to STAT3 and AKT phosphorylation assays, as well as gene and protein expression analyses for fibrosis-related proteins.

### RT-qPCR and PCR

RT-qPCR was performed as previously reported [6], [35]. In brief, total RNA from cultured cells and HF/NF tissue was extracted using an RNA isolation kit (Takara Bio Inc., Shiga, Japan). The purity of the RNA was evaluated by calculating the A260/A280 ratio, aiming for a value of 1.9–2.0. The primer pairs used for gene amplification from the cDNA template were as follows: Col 1, forward 5′-GAGGGCAACAGCAGGTTCACTTA-3′ and reverse 5′-TCAGCACCACCGATGTCCA-3′; Col 3, forward 5′-CCACGGAAACACTGGTGGAC-3′ and reverse 5′-GCCAGCTGCACATCAAGGAC-3′; α-SMA, forward 5′-GACAATGGCTCTGGGCTCTGTAA-3′ and reverse 5′- TGTGCTTCGTCACCCACGTA-3′; and glyceraldehyde 3-phosphate dehydrogenase (GAPDH), forward 5′-GCACCGTCAAGCTGAGAAC-3′ and reverse 5′-TGGTGAAGACGCCAGTGGA-3′. Results from three independent reactions were used to determine the relative expression levels of the target genes, which were normalized against the expression level of the internal loading control, GAPDH.

The IL-10Rα gene was amplified from the template cDNA using the following primers, with underlined *Bgl*II and *Kpn*I restriction sites: upstream, 5′-GCGAGATCTATGCTGCCGTGCCTCGTAGTGC-3′ and downstream, 5′-CAGGGTACCTCACTCACTTGACTGCAGGCTAGAGAT-3′. The PCR products were ligated into the *p*MD18T vector (Takara Bio Inc.) and the correct sequences were confirmed by DNA sequencing.

### Western Blot Analysis

Cells were harvested, washed in PBS, and resuspended in RIPA cell lysis solution (Beyotime Biotech) supplemented with 200 µg/ml phenylmethylsulfonyl fluoride (Boster, Pleasanton, CA, USA), phosphatase inhibitor cocktail (Sigma), and protease inhibitor cocktail (Sigma). Alternatively, HS/NS tissue was washed in PBS and lysed by using TissueLyserII (Qiagen, Düsseldorf, Germany). The protein concentration in each sample was determined using the bicinchoninic acid assay (Pierce, Rockford, IL, USA).

Western blotting was performed as previously described [6], [35], [39]. Briefly, cell lysates containing equal amounts of protein were separated in 7–10% sodium dodecyl sulfate polyacrylamide gels and transferred onto polyvinylidene fluoride membranes at 100 V for 40–90 min. Membranes were blocked with 5% non-fat milk in TBST (Tris buffered saline/0.5% Tween-20) at room temperature for 3–6 h, and then incubated with rabbit polyclonal antibody against IL-10Rα (Epitomics), a rabbit monoclonal antibody against phospho-STAT3 (p-STAT3; Cell Signaling Technology, Danvers, MA, USA), a mouse monoclonal antibody against STAT3 (Cell Signaling Technology), a rabbit monoclonal antibody against phospho-AKT (p-AKT; Cell Signaling Technology), or a rabbit monoclonal antibody against AKT (Cell Signaling Technology) at 4°C overnight.

For the detection of fibrosis, membranes were incubated overnight at 4°C with a rabbit polyclonal antibody against Col 1 (1∶500 dilution; Epitomics), a rabbit polyclonal antibody against Col3 (1∶500 dilution; Epitomics), or a rabbit monoclonal antibody against α-SMA (1∶1000 dilution; Epitomics). Membranes were then washed four times with TBST and incubated with horseradish peroxidase-conjugated secondary antibodies (1∶2500 dilution; Boster) at 37°C for 1 h. The membranes were again washed four times with TBST, and immunoreactive polypeptide bands were detected using enhanced chemiluminescence reagents (Millipore, Billerica, MA, USA). The signal intensity of each polypeptide was quantified after scanning the membrane with an image analyzer (Alpha Innotech, San Leandro, CA, USA). The membrane was stripped of antibodies and then re-probed with a rabbit monoclonal antibody against β-actin (1∶1000 dilution; Cell Signaling Technology), which was used as an internal loading control.

### Statistical Analysis

Quantitative data are expressed as the mean ± standard error of the mean (SEM). Student’s t-test was used to compare data between two groups, and analysis of variance (ANOVA) was used for multiple-group comparisons. *P* values of<0.05 were considered statistically significant.

## Results

### Morphologic and Molecular Characteristics of HS Tissue and HSFs

Histological observation was first carried out on HS and NS tissues. Both H&E and Masson’s trichrome staining showed numerous fibroblasts in the dermis of HS tissue (Fig. S1a–c, g–i), whereas a lower density of fibroblasts was observed in NS tissue (Fig. S1d–f, j–l). The representative fibrotic feature most commonly observed in the HS lesion was the excessive accumulation of collagen resulting from cellular proliferation and keratinization in the epidermis (Fig. S1g–i). While the collagen fiber bundles appeared flexible and organized into a network in the NS tissue (Fig. S1j–l), they appeared as thick bundles in the HS tissue (Fig. S1a–c, S1g–i).

To clarify the differential expression of fibrosis-related genes in HS tissue and primary HSFs, the transcription levels of Col 1, Col 3 and α-SMA were analyzed by RT-qPCR. Col 1, Col 3, and α-SMA transcription levels in the HS samples were significantly higher than those in the corresponding NS samples (*p* = 0.00155, *p* = 0.00856, and *p* = 0.04031, respectively; Fig. S2). The transcription levels in HSFs were also higher than those in NSFs (*p* = 0.04970, *p* = 0.04630, and *p* = 0.00556, respectively; Fig. S2).

### IL-10-Mediated Reduction of Fibrosis in HSFs

We previously reported that IL-10 has inhibitory effects against skin fibrosis by decreasing the excessive deposition of ECM components [6]. Therefore, we next investigated the capacity of IL-10 to attenuate fibrosis-related protein expression in HSFs and NSFs. Western blot analysis revealed that IL-10 significantly attenuated the protein expression levels of Col 1, Col 3, and α-SMA in HSFs (Fig. S3), but not in NSFs (data not shown). These results are consistent with the anti-fibrotic effects of IL-10 previously observed in TGF-β-stimulated NSFs [6].

### IL-10Rα Expression in HS Tissue and HSFs

**Figure 1:**
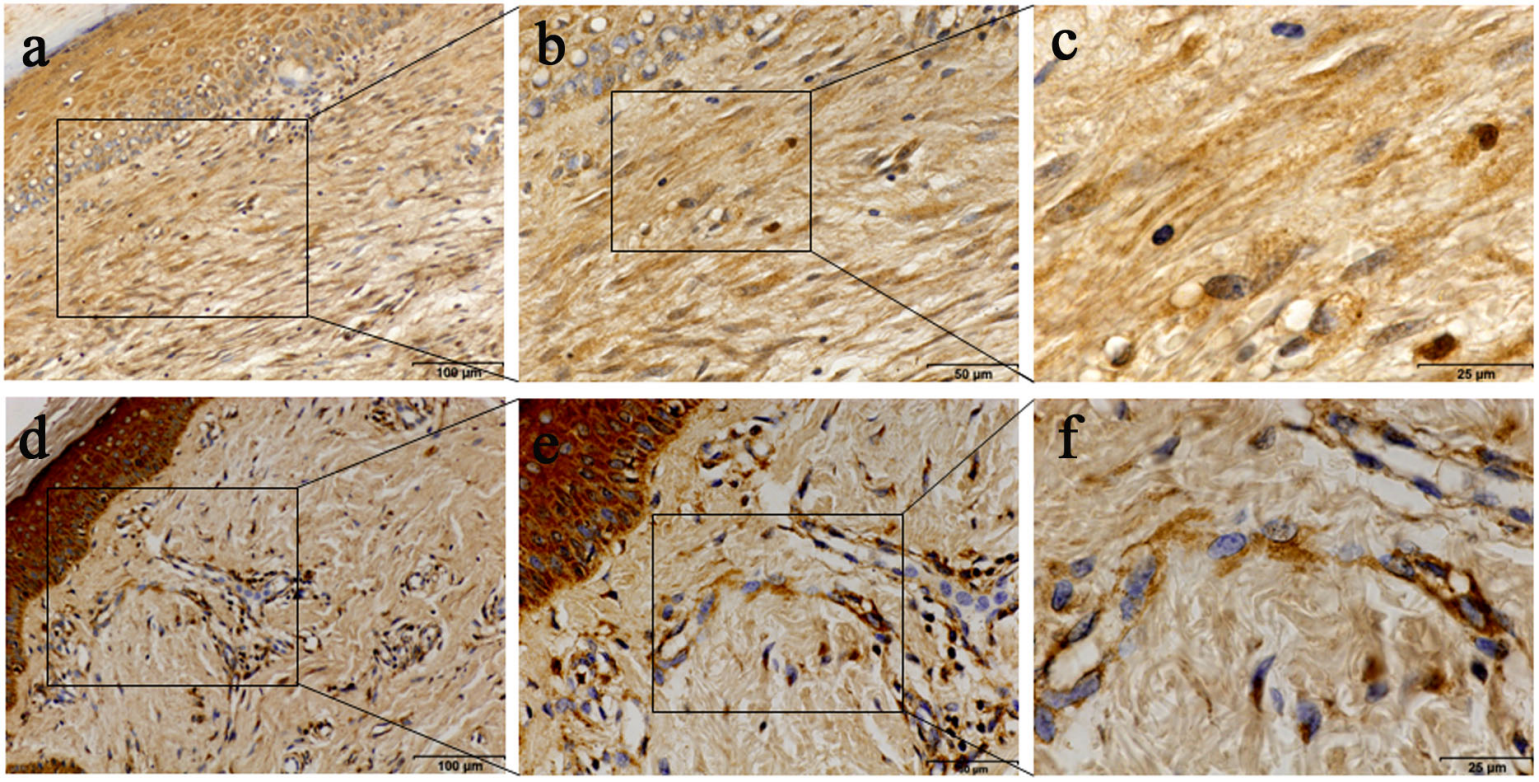
Immunohistochemical analysis of IL-10Rα expression in HS and NS tissue. Streptavidin-peroxidase DAB staining showed that IL-10Rα was localized in HS tissue (a–c) and NS tissue (d–f). IL-10Rα was distributed on the cell membrane and in the cytoplasm, with more intensive staining in NS (d–f) than in HS tissue (a–c). Scale bars, a and d, 100 µm; b and e, 50 µm; c and f, 25 µm.

Immunohistochemistry was performed to assess the expression of IL-10Rα in HSFs. Immunostaining with a specific primary antibody against IL-10Rα showed that the α-chain was localized not only on the cell surface, but also in the cytoplasm of both HS- and NS-derived fibroblasts (Fig. 1). Moreover, the distribution of IL-10Rα in HSFs and NSFs was similar to that in HS and NS tissue (Fig. 1).

**Figure 2:**
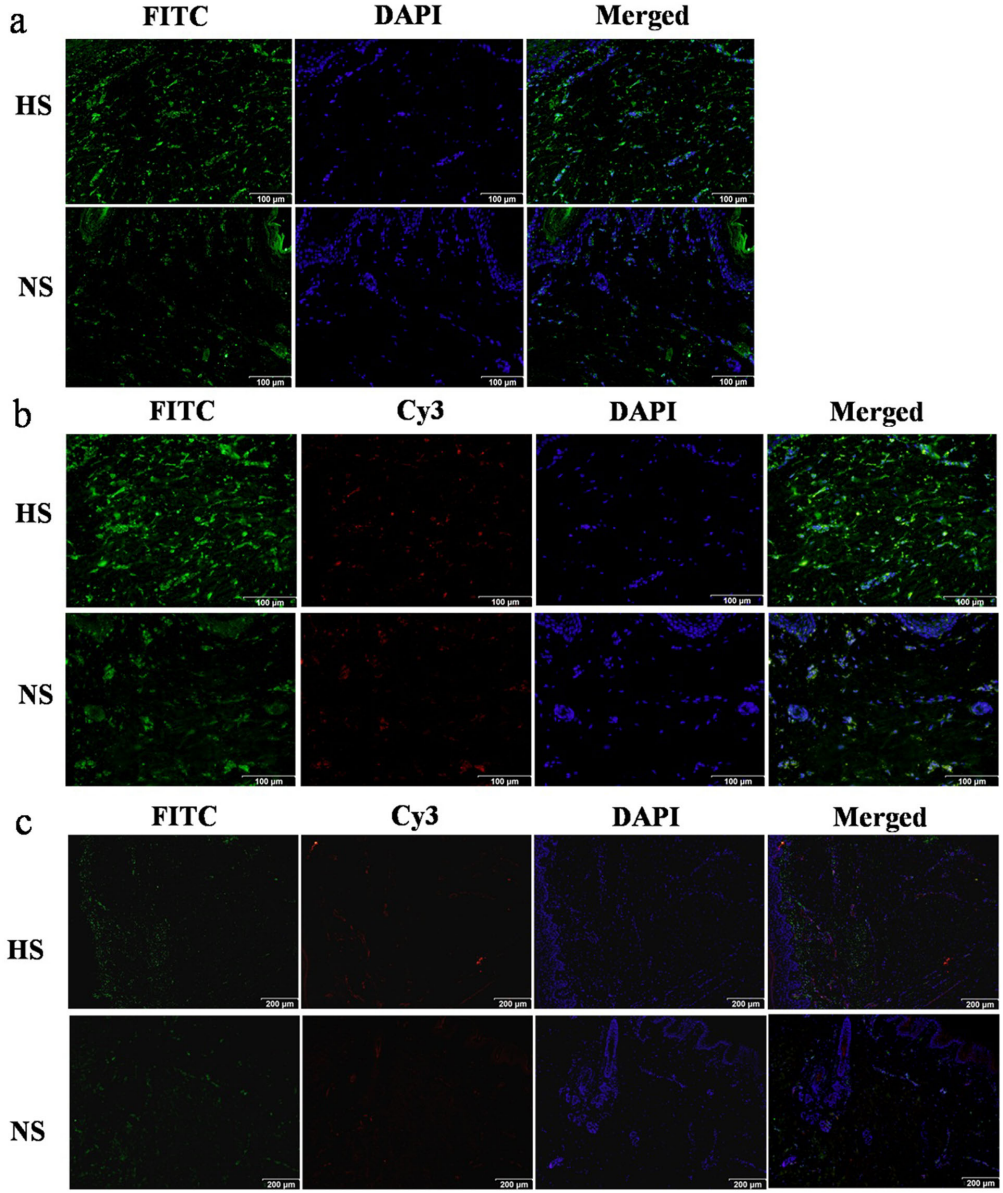
Immunofluorescence analysis of vimentin, CD31, and IL-10Rα expression in HS and NS tissue. (a) Immunofluorescence staining showed that vimentin (green, FITC-conjugated secondary antibody) was localized in HS (upper row) and NS tissue (lower row), with an abundance of vimentin-positive fibroblasts in the HS dermis. Scale bar, 100 µm. (b) Vimentin (green, FITC-conjugated secondary antibody) and IL-10Rα (red, Cy3-conjugated secondary antibody) immunofluorescence staining showed co-localization of both proteins in HSFs (upper row) and NSFs (lower row). Scale bar, 100 µm. (c) CD31 (red, Cy3-conjugated secondary antibody) and IL-10Rα (green, FITC-conjugated secondary antibody) were co-localized on vascular endothelial cells in HS (upper row) and NS tissue (lower row). Scale bar, 200 µm.

To further clarify the cell types that express IL-10Rα in the dermis, we conducted an immunofluorescence analysis for vimentin, CD31, and IL-10Rα. We found that vimentin was distributed in fibroblasts in HS tissue (upper row, Fig. 2a) and NS tissue (lower row, Fig. 2a), with a lower density of vimentin-positive fibroblasts in NS tissue (lower row, Fig. 2a). Co-immunofluorescence staining revealed IL-10Rα expression in vimentin-positive fibroblasts in both tissues (HS, upper row, Fig. 2b; NS, lower row, Fig. 2b), as well as in cluster of differentiation (CD) 31-positive vascular endothelial cells (HS, upper row, Fig. 2c; NS, lower row, Fig. 2c).

**Figure 3:**
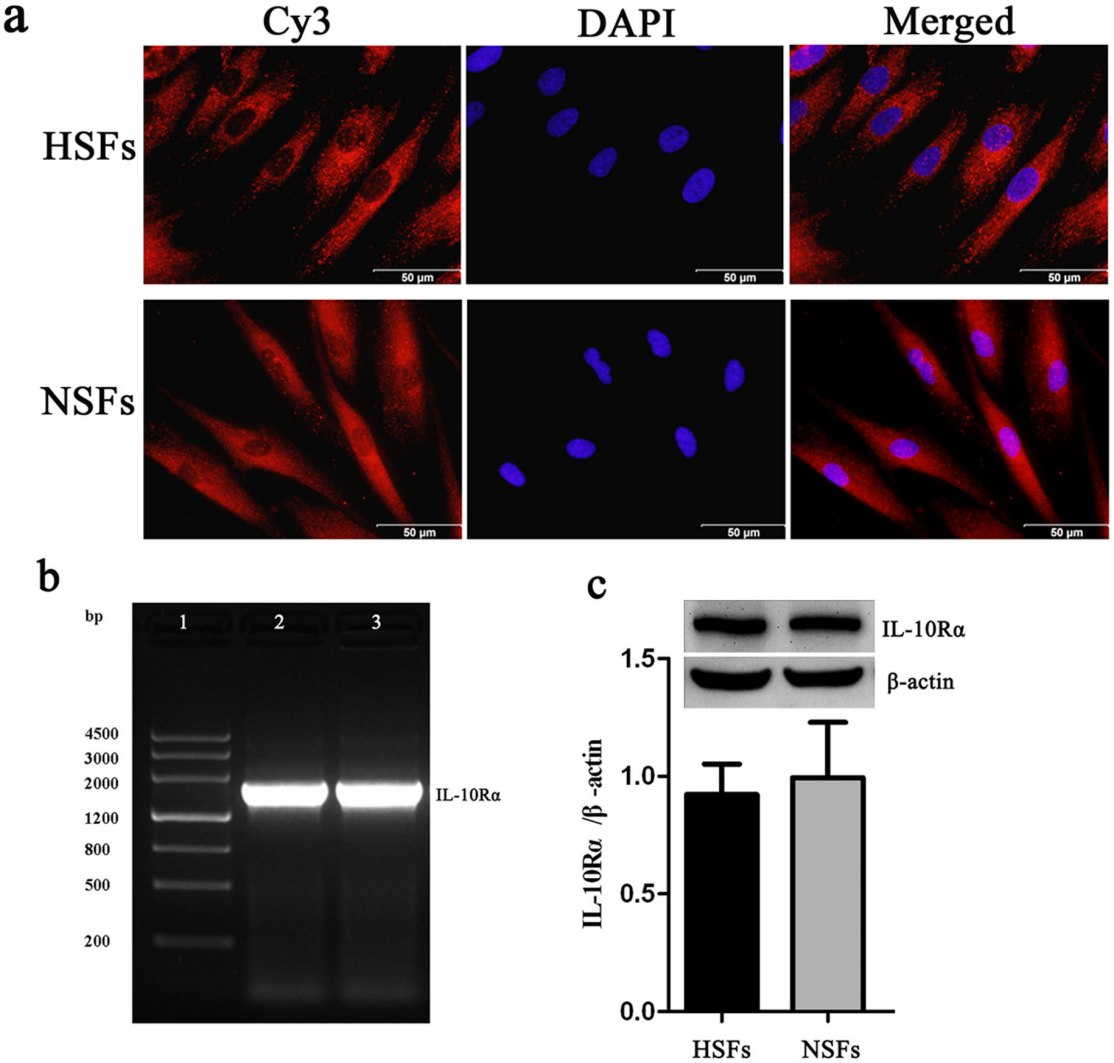
IL-10Rα is expressed by HSFs. (a) HSFs (upper row) and NSFs (lower row) were grown on coverslips until they reached 70–80% confluence, fixed in 10% formaldehyde, washed, permeabilized, and blocked. Cells were incubated with an IL-10Rα monoclonal antibody, followed by incubation with a corresponding Cy3-conjugated secondary antibody. The nuclei of the fibroblasts were stained with DAPI. Scale bars, 50 µm. (b) RT-qPCR was performed to analyze IL-10Rα mRNA expression in HSFs and NSFs. Lane 1, DNA ladder; lane 2, IL-10Rα amplification product in HSFs; lane 3, IL-10Rα amplification product in NSFs. (c) Western blotting was performed to detect IL-10Rα protein (molecular weight, ∼63 kDa) expression in HSFs (hatched bar) and NSFs (closed bar). Data are expressed as the mean ± SEM (*n* = 3, *p*>0.05 *vs.* NSFs).

Immunofluorescence analysis also showed that IL-10Rα was expressed in cultured HSFs (Fig. 3a), while PCR analysis revealed an amplified IL-10Rα transcript (1736 bp) in both HSFs and NSFs (Fig. 3b). The sequence of this amplified fragment was confirmed by Sangon Biotech Co., Ltd. (Sangon, China) and was consistent with that of human IL-10Rα (data not shown). Furthermore, Western blot analysis identified an IL-10Rα polypeptide band with a molecular weight of ∼63 kDa in both cell types (Fig. 3c). Notably, the expression of IL-10Rα protein was lower in HS/HSFs than in NS/NSFs, although the difference was not statistically significant (*p*>0.05, Fig. 3a, c).

### Phosphorylation Levels of AKT and STAT3 in HS/HSFs

**Figure 4:**
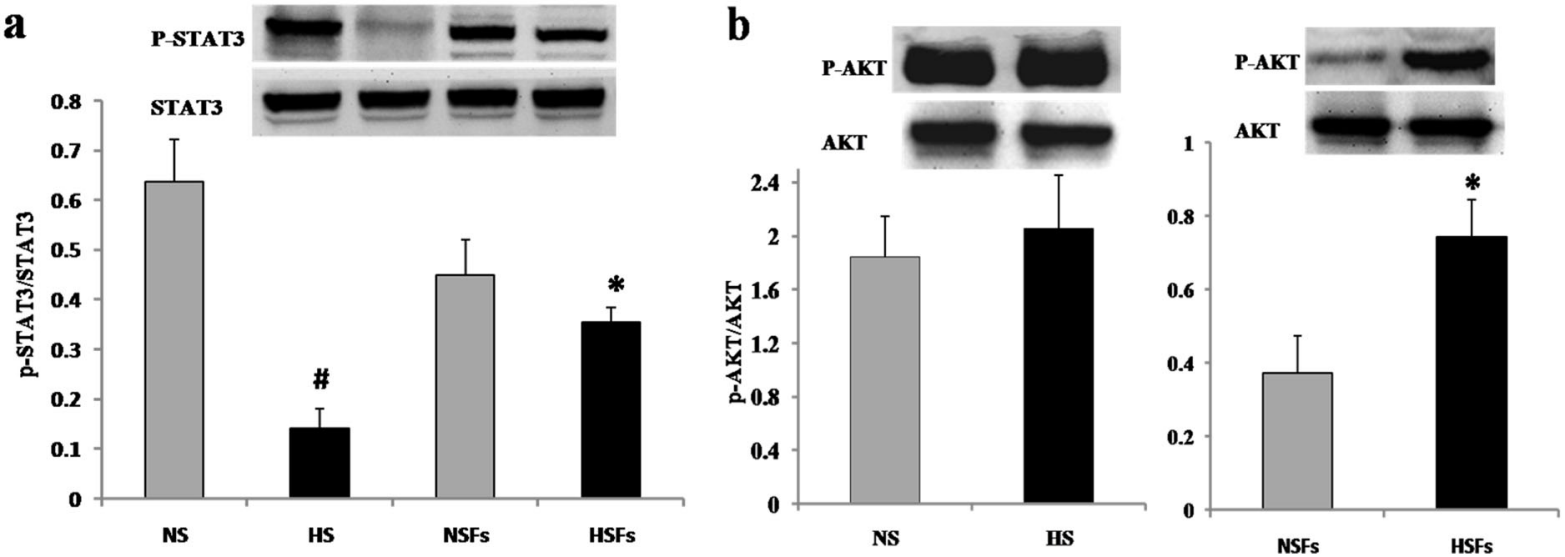
Expression of p-STAT3 and p-AKT in HS/NS tissue and HSFs/NSFs. (a) Expression levels of p-STAT3 were significantly lower in HS than in NS tissue (*p*<0.01) and in lower in HSFs than in NSFs (*p*<0.05). (b) Expression levels of p-AKT were significantly higher in HSFs than in NSFs (*p*<0.05) and tended to be higher in HS than in NS tissue (*p*>0.05). Data are expressed as the mean ± SEM (n = 3, *p<0.05 *vs.* NSFs, ^#^ *p*<0.01 *vs.* NS tissue).

The phosphorylation levels of AKT (p-AKT) and STAT3 (p-STAT3) were determined in HS/NS tissue and HSFs/NSFs. The Western blot results showed that p-AKT levels were significantly higher in HSFs than in NSFs (*p*<0.05, Fig. 4b) and tended to be higher in HS than in NS tissue, although the difference was not statistically significant (*p*>0.05, Fig. 4b). However, p-STAT3 levels were significantly lower in HSFs than in NSFs (*p*<0.05, Fig. 4a) and also lower in HS than in NS tissue (*p*<0.01, Fig. 4a).

### IL-10-Mediated Activation of STAT3 and AKT in HSFs

**Figure 5:**
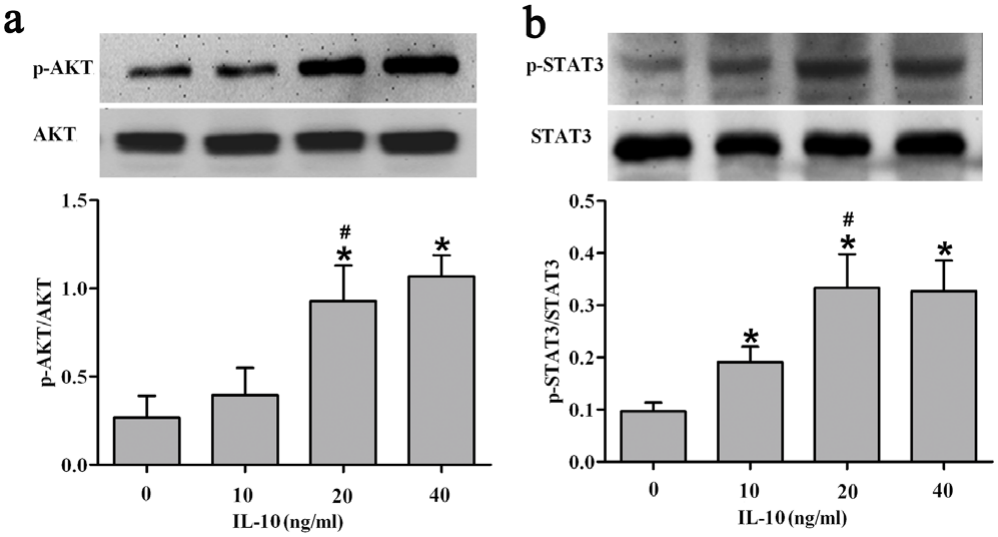
Concentration-dependent phosphorylation of AKT/STAT3 by IL-10 in HSFs. (a, b) Concentration-dependent phosphorylation of AKT (a) and STAT3 (b) in IL-10-treated HSFs. After a 30-min incubation with various doses of IL-10, cells were harvested and analyzed by Western blotting. Data are expressed as the mean ± SEM (*n* = 3, *p<0.05 *vs.* control, ^#^ *p*<0.05 *vs.* HSFs treated with IL-10 at 10 ng/ml).

IL-10 activates the JAK/STAT3 pathway by binding to the IL-10 receptor on the cell surface [24]–[26]. To determine the signaling pathways associated with the inhibition of fibrosis by IL-10 in cultured HSFs, we next examined the impact of IL-10 on the class I PI3K pathway, which is essential for fundamental cellular function [40]–[42]. Consequently, IL-10 dose-dependently increased p-AKT and p-STAT3 levels in cultured HSFs (Fig. 5a, b), demonstrating that IL-10 activates both STAT3 and PI3K/AKT signaling cascades.

**Figure 6:**
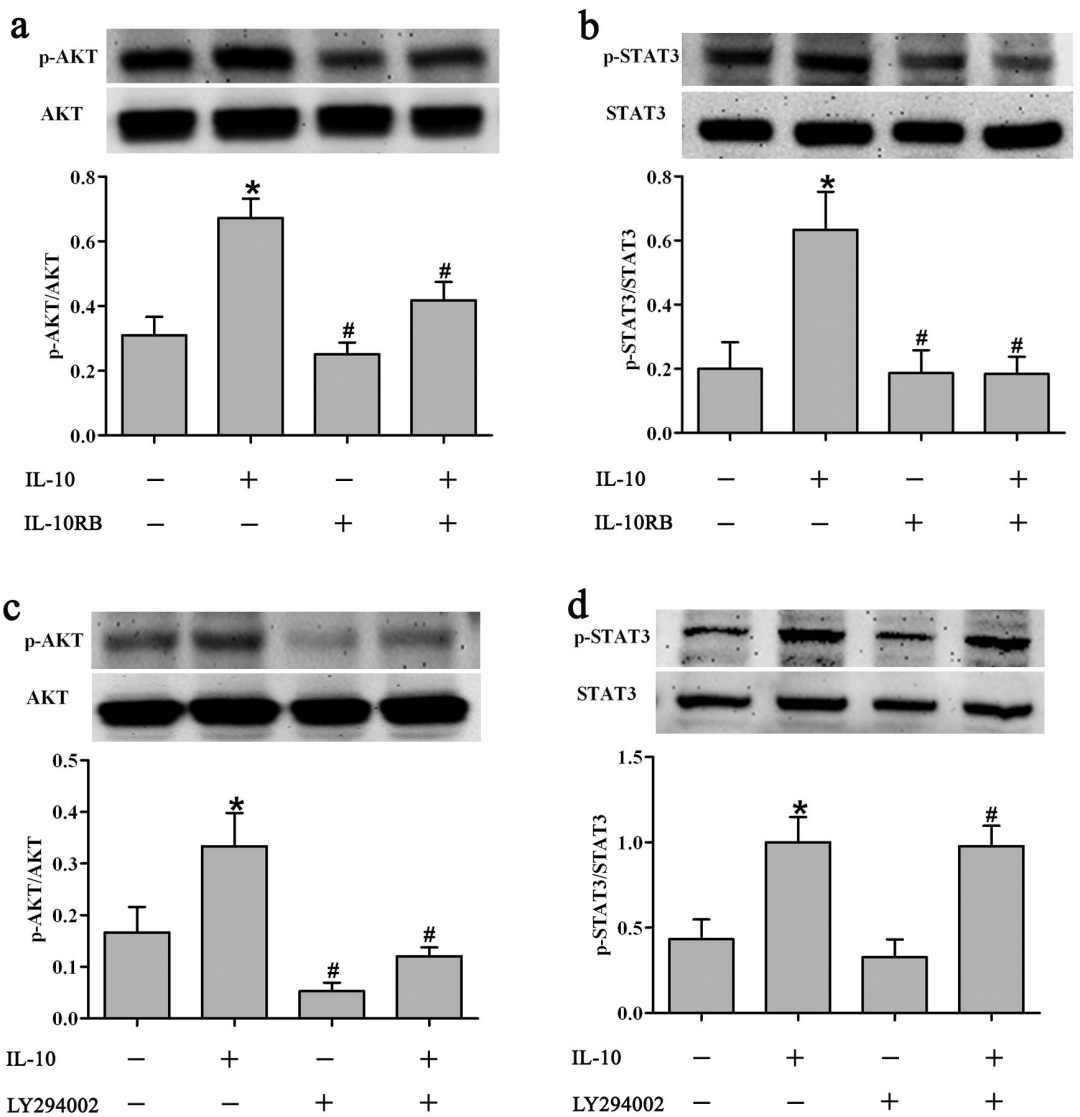
Inhibition of IL-10-mediated AKT and STAT3 phosphorylation in HSFs. (a, b) Inhibition of IL-10-mediated AKT (a) and STAT3 (b) phosphorylation by the function-blocking antibody, IL-10RB (1∶500 dilution), at 30 min after the addition of IL-10 (20 ng/ml), IL-10RB, or both. Data are expressed as the mean ± SEM (*n* = 3, *p<0.05 *vs*. control, ^#^ *p*<0.05 *vs*. HSFs treated with IL-10RB). (c, d) Inhibition of IL-10-mediated AKT (c) and STAT3 (d) phosphorylation by the PI3K inhibitor, LY294002 (50 µM) within 30 min of the addition of IL-10, LY29400, or both. Data are expressed as the mean ± SEM (*n* = 3, *p<0.05 *vs.* control, ^#^ *p*<0.05 *vs.* LY294002-treated HSFs).

To further authenticate these observations, STAT3 and PI3K phosphorylation levels in response to IL-10 treatment were assessed in the presence or absence of a function-blocking antibody against the IL-10 receptor, IL-10RB. IL-10RB reduced PI3K-mediated AKT phosphorylation (*p* = 0.0038, Fig. 6a) and STAT3 phosphorylation (*p* = 0.0340, Fig. 6b) in IL-10-treated HSFs. To confirm the role of PI3K in AKT phosphorylation, we next used a specific PI3K inhibitor, LY294002, and found that LY294002 also inhibited IL-10-mediated AKT phosphorylation in HSFs (*p* = 0.0333, Fig. 6c). Interestingly, LY294002 inhibited STAT3 phosphorylation by itself, while IL-10 increased STAT3 phosphorylation in response to LY294002 in HSFs (*p* = 0.0144, Fig. 6d).

### IL-10 Reduces Fibrosis by Promoting Crosstalk between the PI3K/AKT and STAT3 Pathways

**Figure 7:**
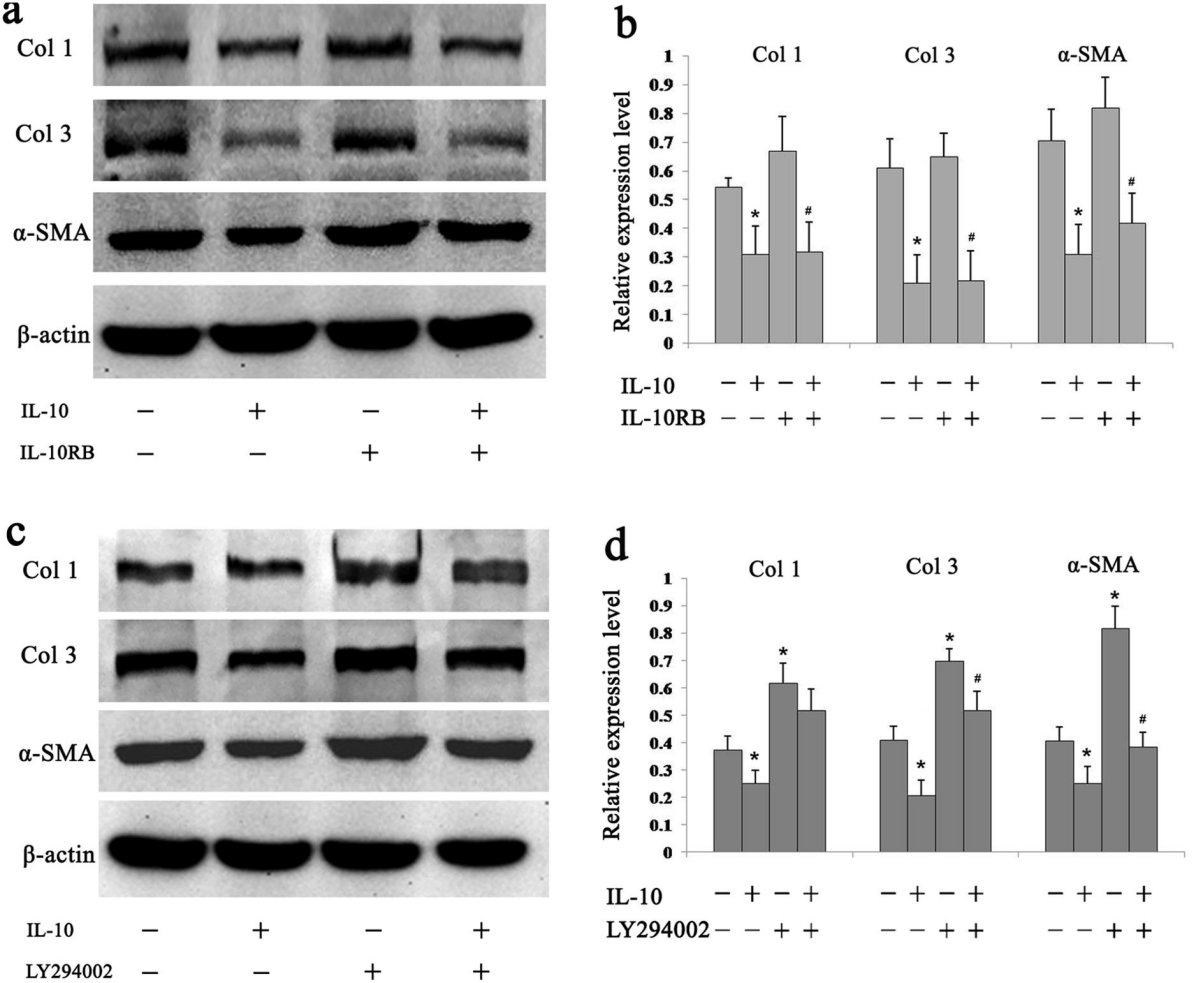
IL-10 reduces fibrosis by activating PI3K and STAT3 pathways in HSFs. (a) Inhibition of IL-10-mediated attenuation of fibrotic markers in HSFs by IL-10RB (1∶500 dilution) at 48 h after the addition of IL-10 (20 ng/ml), IL-10RB, or both. After treatment, cells were harvested and analyzed by Western blotting. (b) Data are expressed as the mean ± SEM (*n* = 3, *p<0.05 *vs.* control, ^#^ *p*<0.05 *vs.* IL-10RB-treated HSFs). (c) Inhibition of IL-10-mediated attenuation of fibrotic markers in HSFs by LY294002 (50 µM) at 48 h after the addition of IL-10 (20 ng/ml), LY294002, or both. After treatment, cells were harvested and analyzed by Western blotting. (d) Data are expressed as the mean ± SEM (*n* = 3, *p<0.05 *vs*. control, ^#^ *p*<0.05 *vs*. LY294002-treated HSFs).

Fibrosis is widely accepted as the main characteristic of HS, and the fibroblast is the major effector cell of fibrosis. To confirm whether IL-10 exerts its anti-fibrotic actions through the activation of PI3K/AKT and STAT-3 signal transduction pathways in cultured HSFs, IL-10RB and LY294002 were individually used to block STAT3 or PI3K phosphorylation for 48 h. As shown in Figure 7a and b, IL-10 significantly down-regulated the expression levels of Col1, Col3, and α-SMA (*p* = 0.03104, *p* = 0.00210, and *p* = 0.01028, respectively). After IL-10RB treatment of IL-10-treated cells, Col1, Col3, and α-SMA expression levels were up-regulated to various degrees, although the differences were not statistically significant (*p*>0.05, Fig. 7a, b). Col1, Col3, and α-SMA expression levels were again significantly down-regulated by IL-10 treatment in another experiment (*p* = 0.04724, *p* = 0.00403 and *p* = 0.04811, respectively; Fig. 7c, d), whereas LY294002 overturned the effects of IL-10 (*p* = 0.07272, *p* = 0.00681, and *p* = 0.00372, respectively; Fig. 7c, d). Therefore, in agreement with studies showing a link between AKT phosphorylation and fibrosis in HSFs, LY294002 reduced the anti-fibrotic actions of IL-10, concomitant with diminished p-AKT levels. Importantly, joint treatment of HSFs with IL-10RB plus LY294002 completely abolished the capacity of IL-10 to mitigate fibrosis (data not shown).

## Discussion

HS formation usually results from abnormal regulation of the tightly-controlled tissue repair mechanism after traumatic skin injury [1], [5]. HS is a significant skin fibrotic condition that exerts a negative impact on patients’ appearance, skeletal muscle function, and general quality of life. It is not only aesthetically displeasing, but also hinders normal function, contributing to psychological as well as physical suffering [3]–[6]. A major feature of HS is the disorganized synthesis of the collagen-based ECM [1], [27], [28], [30], [31]. Therefore, our first step was to evaluate HS and NS skin tissue samples by H&E staining, Masson’s trichrome staining, and RT-qPCR. The results from all of these assays confirmed that the pathological samples had the typical features of HS (Fig. S1, S2).

Currently, there is no effective therapy for HS, largely because the underlying mechanisms of HS development are poorly understood [7], [8]. IL-10 has recently been identified as a promising new therapeutic agent that can reduce HS [16]–[19]. Our previous work showed that IL-10 protects against TGF-β1-induced fibrosis in dermal fibroblasts and can reduce skin scarring [6]. IL-10 down-regulated collagen expression in TGF-β1-treated dermal fibroblasts, up-regulated MMP expression (mainly MMP1 and MMP8), and repressed the transformation of fibroblasts into myofibroblasts, leading to the degradation of excessive ECM components, attenuation of fibrosis, and promotion of scar improvement. These results provide direct evidence that IL-10 can inhibit excessive ECM deposition and suggest that IL-10 plays a novel role in wound healing.

It is generally accepted that IL-10 exerts its biological effects by interacting with a specific cell surface receptor and by stimulating signal transduction [25], [42]. Therefore, we next examined the cellular expression of the IL-10 receptor in HSFs. IL-10Rα expression was detected not only on the cell surface, but also in the cytoplasm of HS/HSFs and NS/NSFs (Fig. 1, 2a, 3a).

Vimentin is a characteristic marker of mesenchymal cells, including fibroblasts, while CD31 is a characteristic marker of vascular endothelial cells. Therefore, the co-localization of vimentin and IL-10Rα, in addition to CD31 and IL-10Rα, was assessed in double immunofluorescence labeling experiments. We found that IL-10Rα co-localized with vimentin in fibroblasts (Fig. 2b) and with CD31 in vascular endothelial cells (Fig. 2c) in HS as well as NS tissue. Furthermore, IL-10Rα was detected in both HSFs and NSFs at the DNA (Fig. 3b) and the protein level (Fig. 3c). Meanwhile, Western blotting data showed that the phosphorylation level of STAT3 was lower in HS vs. NS tissue, and also in HSFs vs. NSFs (Fig. 4a). Interestingly, the phosphorylation level of AKT was higher in HSFs vs. NSFs, but the difference was not statistically significant (Fig. 4b). These findings suggest that p-AKT and p-STAT3 may critically contribute to HS formation.

STAT3 is an essential component of IL-10 signaling and mediates the direct activation of IL-10. STAT3 is phosphorylated by JAK1 and Tyk2, causing STAT3 dimerization and translocation into the nucleus, and inducing expression of its target genes [24]–[26], [43]. AKT, also known as protein kinase B, has emerged as a focal point in signal transduction pathways and is responsible for cell survival, energy metabolism and protein synthesis [41], [42]. Our results showed that IL-10 dose-dependently increased the phosphorylation level of STAT3 at Tyr705 and AKT at Ser473 (Fig. 5a, b). Treatment of HSFs with a function-blocking antibody against the IL-10 receptor, IL-10RB, resulted in the inhibition of STAT3 (Fig. 6a) and AKT (Fig. 6b) phosphorylation, while treatment of HSFs with the PI3K blocker, LY294002, likewise down-regulated AKT (Fig. 6c) and STAT3 (Fig. 6d) phosphorylation. These results imply that IL-10 activates crosstalk between the AKT and STAT3 pathways in HSFs.

To determine whether IL-10 diminishes fibrosis by triggering crosstalk between the PI3K/AKT and STAT3 pathways, IL-10, IL-10RB and LY294002 were applied to cultured HSFs for 48 h. The anti-fibrotic effect of IL-10 was partly inhibited by the antibody and LY294002 when treated individually (Fig. 7a–d), and completely blocked when treated with both reagents together (data not shown). These data further validate the hypothesis that IL-10 inhibits the fibrosis of HSFs by inducing AKT and STAT3 phosphorylation via crosstalk between their associated signaling cascades.

**Figure 8:**
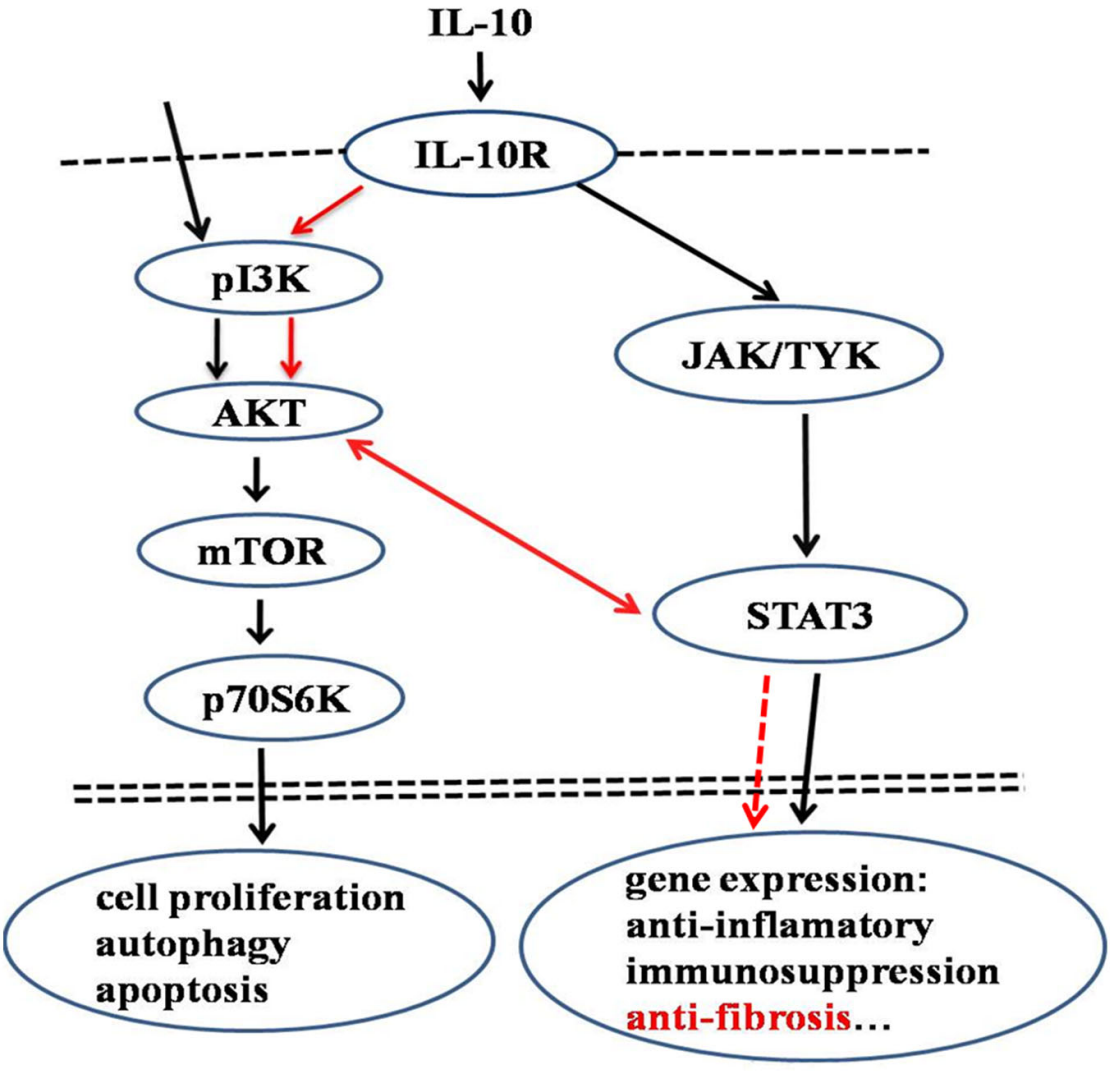
Schematic diagram showing the anti-fibrotic effect of IL-10 mediated by joint activation of the PI3K and STAT3 pathways. IL-10 interacts with its membrane-bound receptor in HSFs to induce class I PI3K/AKT and STAT3 signaling. The stimulation of crosstalk between these signaling pathways results in the inhibition of fibrosis-related gene expression, ultimately leading to diminished fibrosis and scar improvement.

In conclusion, the current investigation proposes a novel anti-fibrotic mechanism of IL-10 in HS tissue. As illustrated schematically in Figure 8, IL-10 protects HSFs against fibrosis via a receptor-mediated mechanism involving the joint activation of AKT and STAT3 signal transduction pathways. Furthermore, the crosstalk between these two signaling pathways effectively blocks the expression of fibrosis-related genes and the excessive deposition of ECM components. Therefore, IL-10 might find therapeutic utility in strategies to reduce fibrotic scarring.

## Supporting Information

Figure S1 **Histological characterization of HS and NS.** (a–f) H&E staining of HS and NS sections. H&E staining revealed a thicker epidermal layer of keratinocytes and a higher dermal cell density in HS (a–c) than those in NS (d–f). (g–l) Massion’s trichrome staining of HS and NS sections. The collagen fibers appeared swirl-shaped in thick bundles in HS (g–i), while flexible and organized in NS (j–l). Scale bars, a, d, g and j, 200 µm. b, e, h and k, 100 µm. c, f, i and l, 50 µm.
(TIF)

Figure S2 **Comparison of Col 1, Col 3 and α-SMA expressions on mRNA level between HS/HSFs and NS/NSFs.** The HS biopsies (hatched bar), NS biopsies (closed bar), HSFs and NSFs were collected and the RNA were extracted from each sample. The mRNA levels of fibrosis-related genes were quantified by qPCR. For all the experiments, mRNA values were normalized against corresponding GAPDH and presented as a ratio to control group (NS/NSFs group, arbitrarily set as 1). Data are expressed as the represents mean ± SEM (*n* = 3, *p<0.05 & **p<0.01 *vs.* the corresponding control).
(TIF)

Figure S3 **Inhibitory effect of IL-10 on the expression of Col1, Col3, and α-SMA in HSF.** HSF was grown to 70–80% confluence and incubated 12–16 h in serum-depleted medium and treated with different dosage of IL-10 (0, 5, 10, 20, 40 and 80 ng/ml) for 48 h. The protein levels of Col1, Col3, and α-SMA were analyzed by Western blot. IL-10 dose-dependently decreased the expression of Col1, Col3, and α-SMA.
(TIF)
