# Supplementary figures and images for: Anti-Fibrotic Actions of Interleukin-10 against Hypertrophic Scarring by Activation of PI3K/AKT and STAT3 Signaling Pathways in Scar-Forming Fibroblasts
Source: PLoS One. 2014 May 30;9(5):e98228. doi: 10.1371/journal.pone.0098228 (PMC4039501; doi:10.1371/journal.pone.0098228)

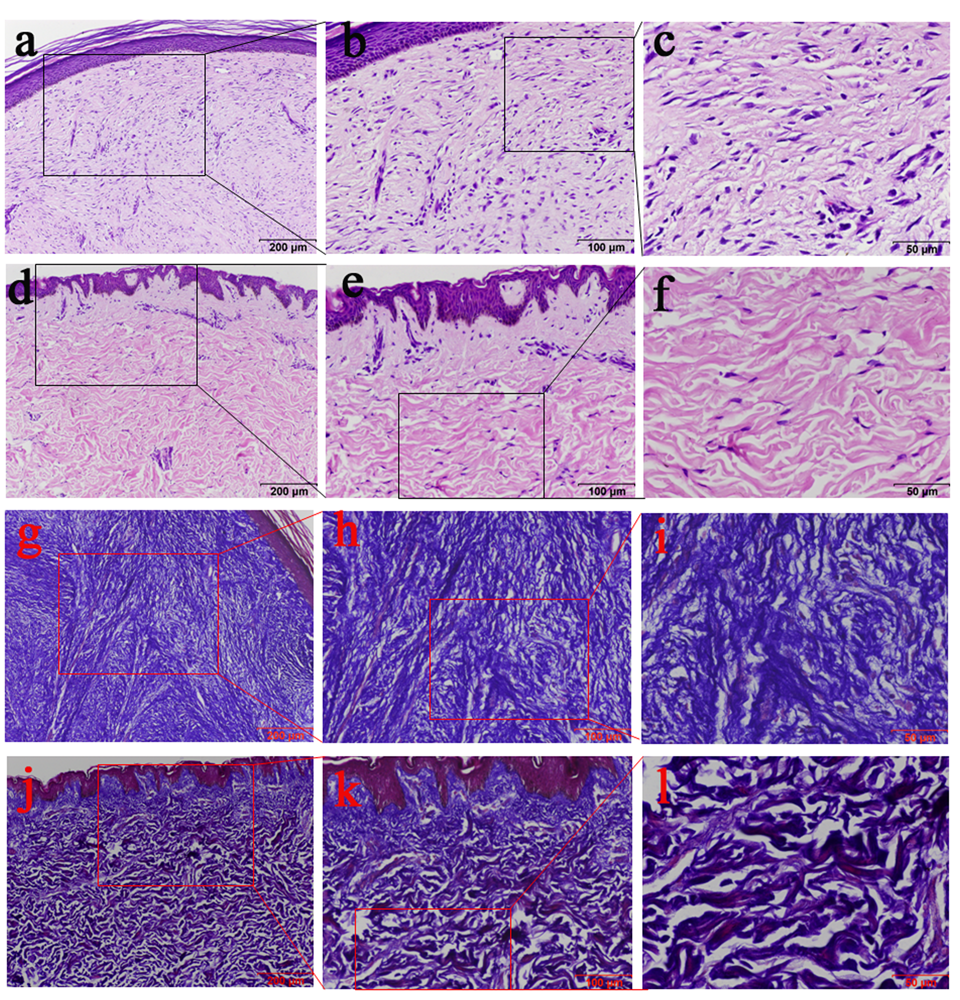

Supplement: Figure S1 — Histological characterization of HS and NS. (a–f) H&E staining of HS and NS sections. H&E staining revealed a thicker epidermal layer of keratinocytes and a higher dermal cell density in HS (a–c) than those in NS (d–f). (g–l) Massion’s trichrome staining of HS and NS sections. The collagen fibers appeared swirl-shaped in thick bundles in HS (g–i), while flexible and organized in NS (j–l). Scale bars, a, d, g and j, 200 µm. b, e, h and k, 100 µm. c, f, i and l, 50 µm. (TIF) [file pone.0098228.s001.tif]

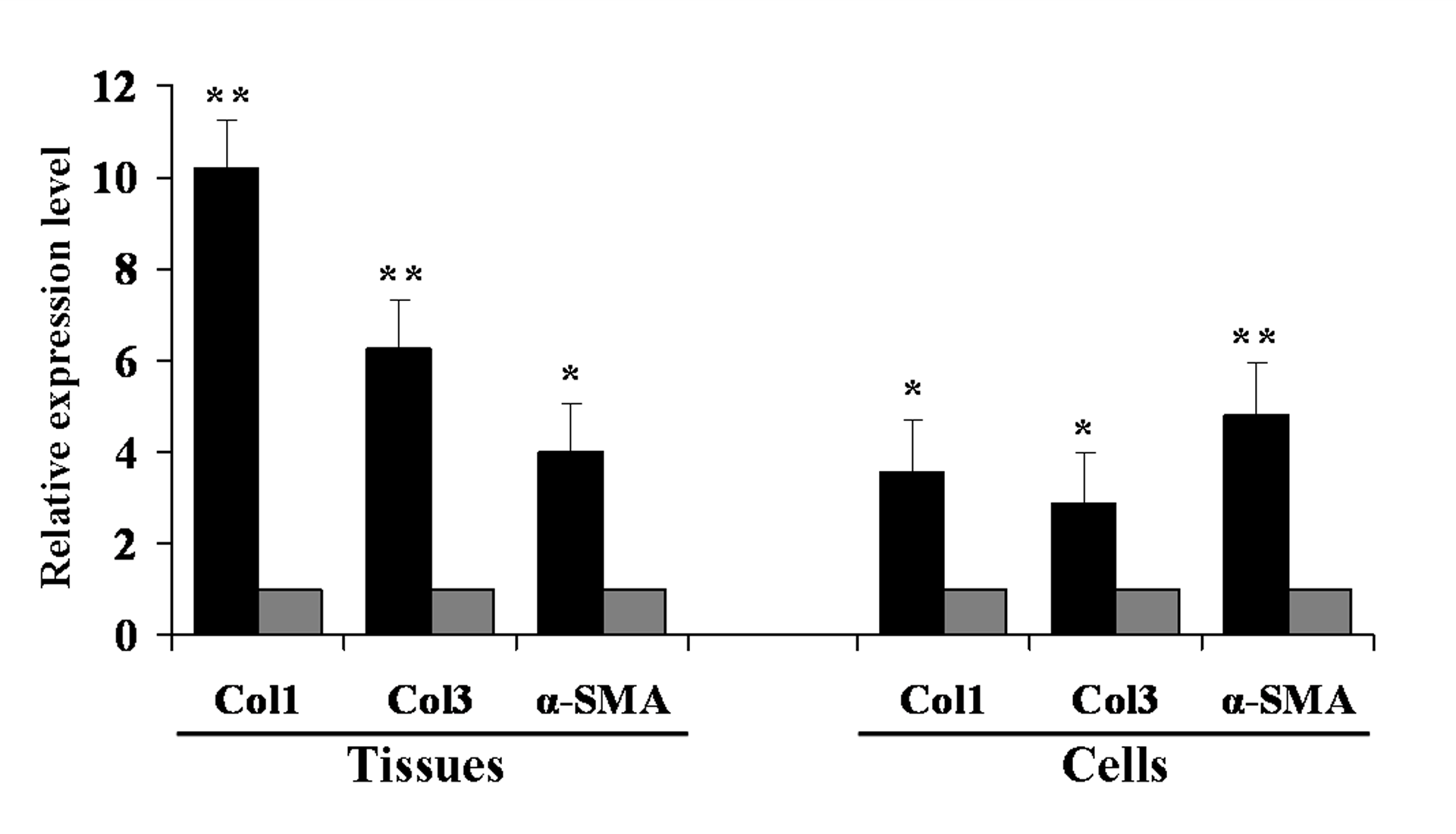

Supplement: Figure S2 — Comparison of Col 1, Col 3 and α-SMA expressions on mRNA level between HS/HSFs and NS/NSFs. The HS biopsies (hatched bar), NS biopsies (closed bar), HSFs and NSFs were collected and the RNA were extracted from each sample. The mRNA levels of fibrosis-related genes were quantified by qPCR. For all the experiments, mRNA values were normalized against corresponding GAPDH and presented as a ratio to control group (NS/NSFs group, arbitrarily set as 1). Data are expressed as the represents mean ± SEM (n = 3, *p<0.05 & **p<0.01 vs. the corresponding control). (TIF) [file pone.0098228.s002.tif]

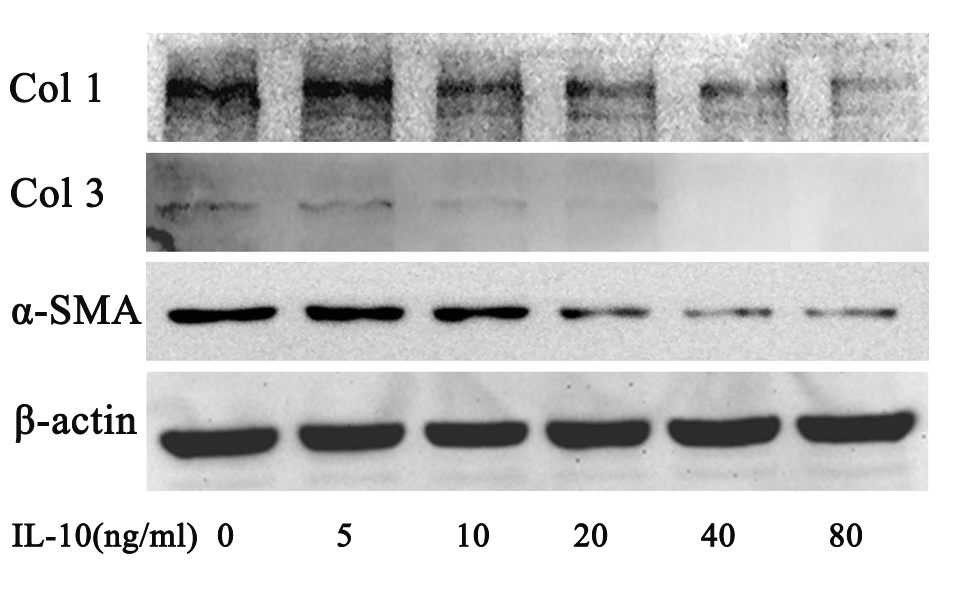

Supplement: Figure S3 — Inhibitory effect of IL-10 on the expression of Col1, Col3, and α-SMA in HSF. HSF was grown to 70–80% confluence and incubated 12–16 h in serum-depleted medium and treated with different dosage of IL-10 (0, 5, 10, 20, 40 and 80 ng/ml) for 48 h. The protein levels of Col1, Col3, and α-SMA were analyzed by Western blot. IL-10 dose-dependently decreased the expression of Col1, Col3, and α-SMA. (TIF) [file pone.0098228.s003.tif]
